# High-Potency Prenatal Cannabis Exposure and Birth Outcome Measures

**DOI:** 10.3390/children11121436

**Published:** 2024-11-26

**Authors:** Natalia M. Kleinhans, Allegra J. Johnson, Sarah F. Larsen, Sara K. Berkelhamer, Mary E. Larimer, Stephen R. Dager

**Affiliations:** 1 Department of Radiology, University of Washington, Seattle, WA 98195, USA; ajj16@uw.edu (A.J.J.); salarsen@uw.edu (S.F.L.); srd@uw.edu (S.R.D.); 2 Integrated Brain Imaging Center, University of Washington, Seattle, WA 98195, USA; 3 Institute on Human Development and Disability, University of Washington, Seattle, WA 98195, USA; 4 Division of Neonatology, Department of Pediatrics, University of Washington, Seattle, WA 98195, USA; berkelsa@uw.edu; 5 School of Medicine, University of Washington, Seattle, WA 98195, USA; larimer@uw.edu; 6 Department of Psychiatry and Behavioral Sciences, University of Washington, Seattle, WA 98195, USA; 7 Department of Psychology, University of Washington, Seattle, WA 98195, USA; 8 Department of Biomedical Engineering, University of Washington, Seattle, WA 98195, USA

**Keywords:** pregnancy, marijuana, THC, sex differences, fetal growth

## Abstract

**Background/Objectives:** Pregnant women have limited information on the impact of prenatal cannabis exposure (PCE) alone. Our aim was to determine if PCE, without alcohol, tobacco, or illicit drug use, is associated with altered birth outcome measures in obstetrically low-risk women. **Methods:** In this observational cohort study, pregnant women were recruited between 2019 and 2022 from communities in Washington and Oregon, USA, and enrolled following their first trimester. PCE eligibility required a minimum of three days/week of cannabis use during the first trimester with no required minimum use thereafter. For all participants, illicit drug, nicotine, or alcohol use was exclusionary throughout pregnancy and monitored via urine toxicology at multiple time points. Cannabis use was quantified into delta-9-tetrahydrocannabinol (THC) and cannabidiol (CBD) mg/day using product weight and potency. Outcome measures included gestational age, weight, length, head circumference, and Apgar scores. **Results:** Study participants included 37 people in the PCE cohort and 35 controls. Average cannabis use for the PCE cohort was 198.0 mg of THC (SD = 221.2 mg)/day and 3.5 mg of CBD (SD = 4.3)/day. PCE newborns weighed less (38th vs. 52nd percentile, *p* = 0.04) and were shorter (40th vs. 55th percentile, *p* = 0.03) for their gestational age than controls. Female PCE newborns had smaller head circumference for gestational age (28th percentile; SD = 23), compared to male PCE newborns (55th percentile; SD = 32; *p* = 0.02). **Conclusions:** PCE is associated with reduced birth weight and shorter length for gestational age. The effect of PCE on brain growth may be sexually dimorphic. Future PCE studies should include sex as a biological variable and longitudinally evaluate long-term developmental and physiological outcomes.

## 1. Introduction

Cannabis use during pregnancy has dramatically increased during the past two decades [1], reflecting, in part, increasing legalization in the United States of America (USA) and growing societal acceptance. Public perspectives are shifting toward regarding cannabis as a benign treatment for pregnancy symptoms and as an alternative to medications that may confer risk to the fetus [2], with pregnant women attempting to balance possible risk of harm to their fetus with decisions about use for their own symptomatic relief. For example, pregnant women are increasingly self-medicating with cannabis for its antiemetic properties to treat “morning sickness”, which is a hallmark of the first trimester of pregnancy and coincides with a critical window for fetal development [2].

Unlike alcohol and nicotine, prenatal cannabis exposure (PCE) has not consistently been associated with fetal growth restriction or fetal pathology. However, as cannabis potency has increased tenfold over the past 40 years [3,4], clinically significant adverse outcomes may be more evident among currently exposed newborns. Research in this area is underdeveloped and challenged by confounders, but well-controlled studies have identified a correlation between PCE and adverse neonatal outcomes [5], such as preterm birth, being small for gestational age (SGA), and admission to the neonatal intensive unit (NICU) [6]. Similarly, a recent meta-analysis that included participants using modern, high-potency cannabis products concluded PCE was associated with greater risk of prematurity, SGA, and perinatal mortality, even after accounting for prenatal tobacco use [7]. However, those authors noted that findings were substantially limited due to internal validity factors, including lack of randomization and various confounders, which are challenges inherent to research on pregnant women [8]. Further, these retrospective studies rely on medical records and cannot effectively quantify cannabis use and assess potency or verify the lack of polysubstance use.

In addition to the impact of higher-potency exposures, the role of sex in vulnerability to PCE remains poorly characterized. Few studies on PCE have tested sex effects on in utero growth or gestational duration. Translational work in a murine model identified reduced placental weight, birth weight, and the fetal-to-placental weight ratio in PCE male but not PCE female pups [9]. Conversely, a large medical records-based retrospective study reported that female PCE newborns were smaller for gestational age and born preterm, after controlling for other substance use and socio-demographic/economic characteristics [6].

This current study evaluated birth outcomes in PCE newborns utilizing a novel approach designed to methodically quantify cannabis use during pregnancy and ensure that infants were not gestationally exposed to alcohol, nicotine, or illicit substances. We enrolled obstetrically healthy participants after the first trimester and carefully conducted multiple drug screens throughout the remainder of their pregnancy. In addition, cannabis consumption was quantified into mg of THC and CBD [10], which allowed us to characterize cannabis use during pregnancy with precision not previously reported. Our aims were to determine whether PCE was related to alterations in birthweight, length, head circumference, Apgar scores, or gestational duration and to assess for relationships with the quantity of cannabis consumed. In addition, we evaluated sex effects on birth outcome measures. Based on the existing literature, we hypothesized that PCE newborns would have a shorter gestation and lower growth parameters, with females more likely to be affected.

## 2. Materials and Methods

### 2.1. Study Design

This study is structured as an observational cohort study comparing in utero cannabis exposed (PCE) and unexposed control infants.

### 2.2. Participants

Participants were recruited in Washington (WA) and Oregon (OR) between 2019 and 2022 via flyers placed near cannabis shops, outreach to naturopathic medical providers, and social media. Participants were included if they were pregnant and between 21 and 40 years of age. Pregnant women in the PCE group were eligible if they used cannabis-containing products purchased exclusively from state-approved dispensaries a minimum of three days per week on average during the first trimester of pregnancy. Once enrolled, PCE participants were not required to continue using cannabis to remain in the study. Pregnant controls were included if they had no self-reported cannabis use history during the current pregnancy, and no more than 5 uses in the year prior to becoming pregnant. All participants were provided with a list of community resources, referrals, and a fact sheet about cannabis use during pregnancy compiled by the Organization for Teratology Information Specialists (www.MotherToBaby.org/fact-sheets/marijuana-pregnancy/ (accessed 1 December 2017).

Potential participants were ineligible for enrollment if they met the following conditions: were undergoing a multiple gestation pregnancy, were younger than 21 (minimum legal age to purchase cannabis in WA and OR); used any substance suspected to impact the health of the fetus (psychotropic or other medications in the first trimester; self-reported illicit drug, nicotine, or alcohol use after the end of week 6); had medical conditions known to impact the health of the fetus (cancer, diabetes, epilepsy, systemic lupus erythematosus, endometriosis, genetic disorder, active neurologic disorder, psychosis, bipolar 1, or a history of suicide attempts (within 2 years of enrollment)); lived with a person who consumed nicotine regularly inside their home; or did not reside in WA or OR.

Once enrolled, any cannabis use by a control while pregnant was exclusionary, as was the use of alcohol, nicotine, or illicit drugs by either PCE or controls for the remainder of the pregnancy (confirmed by toxicology screening). None of our participants tested positive for alcohol or illicit drugs after enrollment. Four participants were excluded after testing positive for cotinine.

Gestational week was based on the participant’s self-reported due date, determined by the date of their last menstrual period or obstetrical dating.

### 2.3. Procedures

This study was designed to enroll participants after their first trimester of pregnancy in order to reduce the chance of spontaneous miscarriage (as the risk of miscarriage decreases after 12 weeks), and, to ensure minimum cannabis use thresholds were met prior to enrollment. Research visit targets were 13 weeks (enrollment), 24 weeks, and 38 weeks with a ±2-week scheduling window. Participants were compensated for their participation, receiving USD 5 per completed weekly diary (week 13 up to 42) and USD 25 for each research visit (up to 3 visits), which consisted of questionnaires and a urine-based drug screen. Participants were compensated up to USD 225 for completing all components of the study.

This study was approved by the University of Washington Human Subjects Division Institutional Review Board, and informed written consent was obtained from all participants.

### 2.4. Data Capture

Study data were collected and managed using REDCap (Research Electronic Data Capture) tools hosted at the Institute of Translational Health Sciences [11,12].

### 2.5. Prenatal Questionnaires and Assessments

#### 2.5.1. Maternal Health and Wellness

Information related to education, employment, income, use of medications, and supplements was collected via questionnaire (Supplementary Materials Table S1). Exercise was characterized based on the Centers for Disease Control and Prevention (CDC) definitions of moderate and vigorous physical activity (exercise levels exceeding 75 min (1 h 15 min) of vigorous activity and/or 150 min (2 h 30 min) of moderate activity) [13]. The Brief Symptom Inventory [14] was used to screen for psychiatric symptoms. The Perceived Stress Scale [15] was used to measure the degree to which participants’ life situations were appraised as stressful. The Pregnancy-Unique Quantification of Emesis [16] was used to assess the severity of symptoms of nausea and vomiting during the previous week. Participants were also asked to report hours slept per night during the previous week.

#### 2.5.2. Maternal Substance Use

At every prenatal visit, participants completed urine drug screening using Rapid Drug Test Cups, an FDA-cleared and CLIA-waived 14-panel drug test cup (testing for THC, cocaine, amphetamine, methamphetamine, phencyclidine, barbiturates, benzodiazepines, methadone, methadone metabolite, MDMA/ecstasy, tricyclic antidepressants, buprenorphine, morphine/opiates, and oxycodone), the SmokeCheck Nicotine Urine Test Cassette, and either the Ethyl Glucuronide (ETG) Rapid Test for alcohol (500 ng/mL) or the Easy@Home alcohol ETG urine test strips (500 ng/mL) to corroborate self-reported lack of polysubstance use.

The Timeline Follow-Back method (TLFB) [17] was administered at the first visit to quantify cannabis use, drug, alcohol, nicotine, and medication use during the first trimester. Anchor dates (e.g., personal events, holidays, estimated conception date, first prenatal visit date) were used to improve retrospective recall and identify important characteristics of cannabis use. Due to pandemic-related disruptions, some participants were recruited later in their pregnancies (beyond 15 weeks). In these cases, the TLFB method was used to measure frequency of cannabis use up to study enrollment [11 PCE (Mean = 22.1, SD = 7.2 weeks) and 12 control (Mean = 26.2, SD = 5.1 weeks)].

Because in-person research visits were suspended in March 2020, we modified our protocol to allow urine screens to be conducted remotely. Participants were mailed the drug screen kit, and a research staff member provided instructions over the phone to ensure valid screening. Participants took photos of the tests and uploaded these photos in real-time to a secure, REDCap survey for a research staff member to read the results. [*n* = 17 (11%) of drug screens were conducted remotely]. Drug screens prior to COVID-19 or after institutional approval for critical research operations to resume in July 2020 were conducted in-person [*n* = 131 (89%) of drug screens].

#### 2.5.3. Cannabis Assessments

After enrollment, PCE participants completed weekly cannabis use diaries during their second and third trimesters [10]. Participants received the first weekly survey link within one week of enrollment and each week thereafter. Our REDCap survey probed for the frequency of cannabis use, product format, mode of consumption (inhale, ingest orally, or topically), amount consumed, brand name, THC/CBD percentages, and pictures of packaging for verification, as previously described. The Marijuana Craving Questionnaire-Short Form [18] and the Cannabis Use Disorder Identification Test [19] were collected to characterize the risk of cannabis addiction in our sample.

### 2.6. Cannabis Quantification

#### 2.6.1. Frequency of Cannabis Use Quantification

Cannabis use data for all participants was aligned to the onset of the last monthly period (LMP, week 0, day 1) and extended through the date of birth. Calculation of day/week use frequency from diary data was determined based on non-zero reported use of either THC or CBD on a given day, as shown in Equation (1).

(1) $$Diary\;Cannabis\;Use\;Frequency\frac{days}{week}=\;\frac{Number\;of\;days\;Used}{Total\;Reported\;Days}\times 7\frac{days}{week}$$

Cannabis use frequency prior to enrollment was captured using the TLFB method starting from LMP. When overlap between diary and TLFB frequency data occurred, the measure with the most complete data was preferentially used, typically TLFB.

#### 2.6.2. Quantification of THC and CBD in Mg

##### Calculating Potency of Cannabis Products

**Figure 1:**
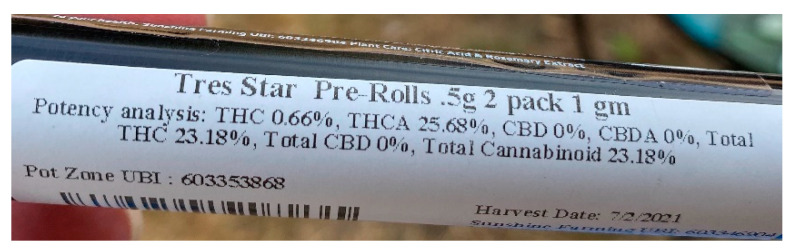
Sample of submitted packaging.

Potency information was obtained from submitted cannabis packaging. Cannabis package labeling is regulated by WA State, requiring net weight, trade name, and concentration of THC, THCA, and/or Total THC and CBD, CBDA, and/or Total CBD (see Figure 1 for an example). THCA and CBDA are cannabinoids converted to THC and CBD via exposure to heat, resulting in decarboxylation. This process is only 87.7% efficient; thus, the formula to compute Total THC% and Total CBD% are shown in Equations (2) and (3) [20].

(2) Total THC %=THC% + 0.877×THCA%

(3) Total CBD %=CBD% + 0.877×CBDA%

##### Estimating the Quantity of Cannabis Product That Was Consumed

For flower, hash, and concentrates, participants were asked to report the net weight of the product that they consumed over an entire day. To provide the flexibility required to quantify the wide range of formats consumed, entry of text strings was allowed for use amounts, with the following conversion factors applied: 1 dab = 0.1 g; 1 puff = 0.0033 g; 1 pre-roll = 0.5 g; 1 joint = 0.5 g; 1 hit = 0.025 g; 1 bowl = 0.25 g. Products recorded as “other”, typically topicals, creams, and gels, were excluded from analysis.

##### Converting the Quantity of Cannabis Consumed into Mg THC and CBD

For flower, concentrate, and hash, this was calculated using the net weight of the amount of cannabis consumed and the THC potency, as captured by Total THC% content per Equation (4). For edibles and tinctures, mg THC was calculated per Equation (5).

(4) $$THCmg=\;Cannabis\;Consumed\;\left(g\right)\times Total\;THC\%\times \frac{1000\;mg}{g}$$

(5) $$THCmg=Number\;of\;Servings\times THC\;\frac{mg}{serving}$$

For example, Figure 1 depicts the label from a 0.5 mg pre-rolled joint with a Total THC potency of 23.18%. Its THC content in mg, calculated per Equation (4), equals 115.9 mg. See Equation (6).

(6) $$THC\;Content\;=0.5\;g\times \left(\frac{1000\;mg}{1\;g}\right)\times 0.2318\;=115.9\;mg$$

Once THC mg is determined for all formats, the amount is summed for each day, regardless of format or route of consumption. This same approach is applied to CBD mg. Bio-availability, which varies by format and consumption route, participants’ prior use history, metabolic factors, genetic makeup, and other factors, is not factored into our calculation, in line with the recently adopted NIDA reporting standard [21].

### 2.7. Postnatal Measures

Birth outcome data (weight (oz), length (cm), head circumference (cm), gestational age at birth, Apgar scores, neonatal intensive care unit (NICU) admission) were obtained directly from mothers or via medical records. PediTools [22] was used to transform raw birth weight, birth length, and head circumference data into percentiles on the Fenton Growth Curve [23]. Birth outcome data were assessed to identify the presence of clinically significant adverse birth outcomes. Newborns who weighed below the 10th percentile for their gestational age were categorized as small for gestational age (SGA) [24]. Newborns who weighed less than 2500 g at birth were categorized as low birth weight (LBW), and newborns born before the 37th week of pregnancy were categorized as preterm, per current CDC definitions [25]. Fetal death occurring before the 20th week of pregnancy was classified as a miscarriage and as a stillbirth after the 20th week [26].

Information on risk factors and complications that occurred during this pregnancy and prior pregnancies was obtained from participants using our Pregnancy History Questionnaire. (Supplementary Materials Table S2). Participants were asked if they experienced any of the following conditions: hypertension, preeclampsia, gestational diabetes, hyperemesis gravidarum, preterm birth, or placental abruption. In addition, we collected information on prenatal care, gravidity, height, and pre-pregnancy weight.

### 2.8. Statistical Analyses

All statistical analyses were completed using SPSS Version 29. Between-group differences and group by sex of newborn interaction effects were tested using multivariate analysis of variance. Follow-up analyses were tested with independent-sample *t*-tests. A paired samples *t*-test was used to test whether participants’ use frequency changed between the first trimester and second and third trimester. Fisher’s exact test was performed on categorical outcome variables. Pearson correlation analyses were conducted to explore relationships between calculated THC and CBD consumption and birth outcome measures. Missing data—for any reason, including lost to contact—were excluded from analysis. Sensitivity analyses were conducted for participants who fell below initial enrollment use quantities.

## 3. Results

### 3.1. Participant Enrollment

**Figure 2:**
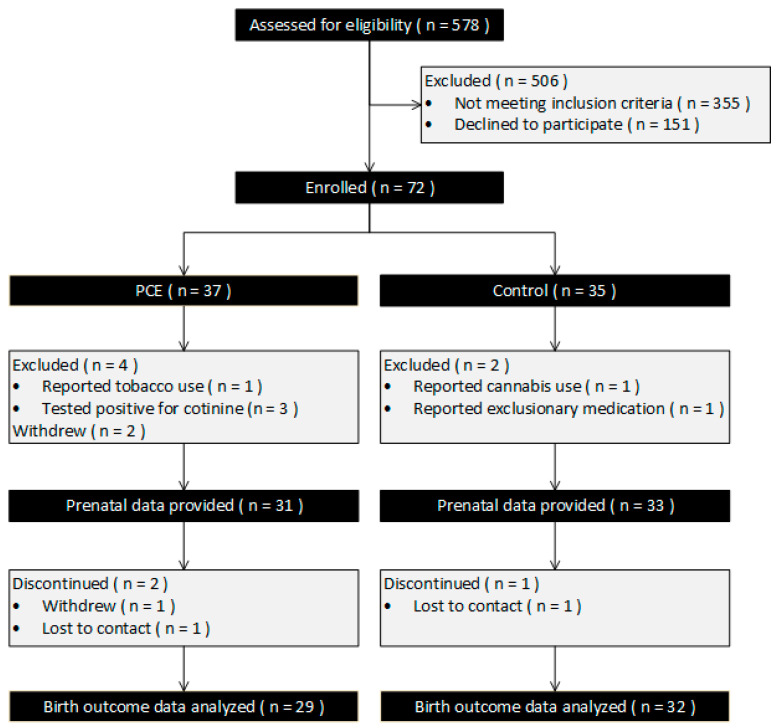
Subject disposition flow diagram.

Of the N = 72 participants enrolled, 31 PCE and 33 controls provided usable data. Details on the recruitment and retention of the study cohort are diagramed in Figure 2, and cohort demographics and pregnancy risk factors are detailed in Table 1. Demographics further segregated by infant sex are detailed in Appendix A Table A1. Research visits were conducted at 17.0 (6.7)-, 27.0 (2.9)-, and 37.8 (0.6)-weeks’ gestation (mean (SD)).

### 3.2. Average Daily Consumption and Frequency of Cannabis Use

PCE participants used cannabis 6.3 days/week (SD = 1.3) on average during the first trimester. During the second and third trimesters of pregnancy, use frequency declined to 5.5 days/week (SD = 1.9; *p* = 0.03), Table 2. Three participants reduced their use to fewer than 3 days/week during the second and third trimester. The average daily quantity of cannabinoids consumed following enrollment, calculated based on diary data, was 198.0 mg/d (SD = 221.2; range = 0.6–745.6) of THC and 3.5 mg/d (SD = 4.3; range = 0.6–16.3) of CBD (Table 2; see Appendix A Table A2 for individual use quantities broken down by format). Products used consisted of flower with a mean THC potency of 20.0% (SD = 10.4), concentrates with a mean potency of 70.4% (SD = 20.9), and edible products, which are sold prepackaged in up to 10 mg serving units.

### 3.3. Group Differences in Birth Outcome Measures

Group statistics for categorical birth outcome measures and continuous birth outcome measures are reported in Table 3. Complete data sets were not available for all participants, which led to unequal sample sizes across our outcome measures.

No significant group differences were observed in the probability of a clinically significant adverse birth outcomes, including miscarriage, stillbirth, SGA, LBW, prematurity, or NICU admission (Table 3, Categorical Birth Outcome Measures). One PCE participant had a miscarriage (sex unknown), and one control participant had a stillbirth (male). One PCE newborn was SGA (female), and another PCE newborn was LBW (male). One control newborn was preterm (female). Sixteen percent (4/25, 1 female, 3 males) of PCE newborns were admitted to the NICU, as opposed to 3% (1/31, female) of control newborns. PCE newborns had comparable weight and length as control newborns; however, when evaluating growth percentile for gestational age, PCE newborns were at the 38th percentile for weight (SD = 24.6; t55 = −2.13, *p* = 0.04) and the 40th percentile for length (SD = 26.9; t55 = −2.21, *p* = 0.03), whereas controls were at the 52nd (SD = 22.8) and 55th percentile (SD = 26.4), respectively. No group differences were observed for gestational age, Apgar scores, or head circumference percentile for gestational age (see Table 3, Continuous Birth Outcome Measures).

When the PCE group was restricted to the subset of newborns whose mother continued to use cannabis products at least 3 days per week throughout pregnancy (*n* = 25), the effect size for weight percentile for gestational age increased from Cohen’s d −0.567 to −0.743 and from Cohen’s d −0.587 to −0.652 for length percentile for gestational age. (Table 4).

### 3.4. Sex Differences in Birth Outcomes

Multivariate analysis of variance was conducted with group (PCE, control), sex of the newborn (male, female), and the interaction between group and sex of the newborn as independent variables and six birth outcome measures (length percentile, weight percentile, head circumference percentile, gestational age at birth, 1 and 5 min Apgar scores). A significant multivariate effect was found for the interaction between group and sex of the newborn (Pillai’s Trace = 0.298, F(6, 44) = 3.11, *p* = 0.01) but not for the main effects of group (*p* = 0.08) or sex (*p* = 0.32). Univariate analyses found a significant interaction effect for newborn weight percentile (F(1, 49) = 7.11, *p* = 0.01) and newborn head circumference percentile (F(1, 49) = 4.99, *p* = 0.03). Significant interaction effects were not found for length percentile, gestational age at birth, or Apgar scores. Sex-specific *t*-tests of PCE newborns identified that the head circumference percentile for gestational age was lower in females compared to males (28th percentile vs. 55th percentile; t23 = 2.43, *p* = 0.02). No sex differences within the PCE group were observed for birth weight or length percentile, gestational age at birth, or Apgar scores (Table 5). Within the non-PCE control group, female newborns were at a higher birth weight percentile than males (63rd vs. 40th; t29 = −3.22, *p* = 0.003). No control group sex differences were found for length, head circumference, Apgar scores, or gestational age at birth.

Factors of socioeconomic status, stress, and psychiatric well being were not significantly correlated (*p* > 0.05) with birth outcomes in our PCE sample (Appendix A Table A3).

## 4. Discussion

We prospectively studied obstetrically low-risk women who had near daily use of high-potency cannabis during pregnancy, without concomitant alcohol, nicotine, or other drug use, confirmed by serial drug testing, to systematically assess cannabis use and relationships to postnatal outcomes in comparison to a non-PCE control group. Accurate characterization of daily cannabis use, separately quantified as mg of THC and CBD, is critical for understanding dose–response relationships within and across studies. Average daily THC consumption in our study was high (i.e., 198 mg/day), 40 times the 5 mg standard unit adopted by the National Institute of Drug Abuse, [27,28], reflecting the current prevalence and popularity of high-potency products available in the retail marketplace. Note that this level of THC consumption can be reached by smoking two 0.5 mg pre-rolled joints per day, which is the most popular size of commercially available pre-rolled joints. Thirty years ago, at 2% Total THC [4], a 0.5 mg joint would have only contained 10 mg of THC, and a comparable exposure would mean smoking 20 joints daily.

While PCE was associated with smaller than expected newborns, differences were modest, and most participants’ birth outcome measurements did not raise significant concerns for long-term developmental and metabolic sequelae associated with in utero growth restriction. Differences were not observed for other birth outcome measures, including gestational age and Apgar scores. Lower birth weight is one of the most replicated adverse neonatal outcomes associated with PCE [29]. However, inconsistent findings have been reported in meta-analysis studies, with at least two reporting that there were no significant differences in birth weight after controlling for tobacco use and other confounding factors [7]. It is possible that a significant PCE effect was observed in this small sample because study participants were users of modern high-potency cannabis products, with over half of the participants consuming more than 100 mg/day of THC throughout the second and third trimesters of pregnancy (Table 2, Appendix A Table A2). Larger prospective samples, encompassing a wider range of cannabis dosage and timing during pregnancy, may help to establish whether THC exposure thresholds, or windows of susceptibility, exist for clinically significant growth restriction and identify co-occurring factors that may interact with PCE to increase post-natal outcome risks.

Results from our study support limited prior research that sex may be an important factor in assessing the risk of adverse birth outcomes associated with PCE. Head circumference showed a large sex effect in PCE newborns, indicating that head circumference in PCE females is reduced (Table 5), whereas head-sparing growth restriction is observed in PCE males. The impact of gestational cannabis use on head growth is unclear. Some studies have identified compromised head growth associated with PCE [30], while others have not seen differences in head size at birth [7]. Sex-related differences in head size in PCE newborns have not been previously reported, raising the possibility that the relationship between cannabis exposure and head size is masked when sex is not accounted for. Larger cohorts or a reanalysis of previously published work would be instrumental in discerning differential effects of PCE on both head growth and developmental outcomes in males and females. Male and female fetuses are known to respond differently to adverse in utero environments [31], yet the literature on sex-based vulnerability to PCE is inconsistent and underdeveloped. Although it is widely accepted that male fetuses are at a greater risk of morbidity and mortality during complicated pregnancies, a growing number of studies, including our own, provide a more nuanced perspective, indicating that females may be differentially impacted. For example, a pre-clinical study in guinea pigs suggest that fetal growth restriction differentially increases neuroinflammatory markers in female compared to male fetuses [32]. Further, in human pregnancies complicated by asthma, the placenta adapts to maintain male fetal growth, whereas there is compromised fetal growth in females, which may serve as a model for conceptualizing our preliminary findings [33].

### Limitations

The main limitations of this study are its small sample size and inconsistent group matching. Factors of socioeconomic status, stress, and psychiatric well being, however, were not correlated with birth outcomes in our PCE sample and are thus unlikely to have driven the group differences reported here (Appendix A Table A3). Participants were systematically drug-tested after study enrollment, but polysubstance use during the first trimester was solely verified by self-report. We also acknowledge that additional factors, which may have impacted fetal growth outcomes—for example, caffeine consumption [34]—were not assessed. Lastly, the route of cannabis use (ingestion versus smoking) was not analyzed. Smoking cannabis generates high levels of absorbed carbon monoxide [35], adversely impacting oxygen transfer from maternal hemoglobin, compromising fetal tissue oxygenation and in utero growth.

## 5. Conclusions

Obstetrically low-risk pregnant women who regularly use high-potency cannabis products, absent concomitant drug, alcohol, or nicotine use, have smaller than expected newborns, with lower newborn birth weight and birth length than non-exposed controls. Female PCE newborns, specifically, may have a smaller head size, as well. Systematically determining the amount of THC and CBD consumed during pregnancy is an important first step towards determining the bioavailable quantity of THC/CBD that enters the blood stream and ultimately crosses the placenta to induce physiological effects on the fetus. Additional research in this area is required to improve the precision of our measurements and provide information on dose–response relationships. The small sample size and group demographic differences were limitations of this preliminary study. Larger samples, encompassing a wider range of cannabis dosage and the route and timing of exposure during pregnancy may help to better establish whether THC exposure thresholds, or windows of susceptibility, exist for clinically significant growth restriction and identify co-occurring factors that may interact with PCE to increase post-natal risks.

## Figures and Tables

**Table 1:** Demographic characteristics and risk factors of enrolled participants.

	PCE, *n* = 31	CON, *n* = 33
Demographics, mean (SD)		
Maternal age at LMP, years	27.9 (3.8)	29.7 (3.2)
Maternal education, years	13.6 (2.0)	16.2 (2.1)
Annual household income, USD	63,151 (42,039)	139,361 (82,796)
Gravidity, No.	2.6 ^a^ (2.3)	1.6 (0.9)
Pre-pregnancy BMI	32.4 ^a^ (9.6)	26.7 (5.1)
Race and ethnicity, No. (%)		
Hispanic	7 (23)	8 (24)
Caucasian	22 (71)	28 (85)
More than one race	2 (6)	1 (3)
African American	1 (3)	1 (3)
Asian	1 (3)	0 (0)
Other ^d^	4 (13)	3 (9)
Declined to respond	1 (3)	0 (0)
Pregnancy risk factors ^a^, No. (%)		
Lack of prenatal care	0 (0)	0 (0)
Hypertension	2 (7)	3 (9)
Preeclampsia	0 (0)	1 (3)
Gestational diabetes	3 (11)	2 (6)
Hyperemesis gravidarum	6 (22)	0 (0)
Previous preterm births	1 (4)	2 (6)
Placental abruption	0 (0)	0 (0)
Exercise ^h^, no. (%)		
First trimester, yes	16 ^e^ (70)	26 ^c^ (84)
Second trimester, yes	18 ^e^ (78)	26 ^c^ (84)
Third trimester, yes	13 ^e^ (57)	21 ^c^ (68)
Medication and supplements, No. (%)		
Prenatal vitamins or folic acid	27 (87)	30 (91)
Prescription antiemetic medication	16 (52)	14 (42)
Mental health and well being, mean (SD)		
BSI GSI T-score	59.0 (8.2)	52.2 (8.3)
Perceived stress scale total	19.8 ^f^ (7.5)	14.4 ^c^ (5.5)
PUQE	5.9 (1.7)	4.1 (1.1)
Sleep, hours/night	6.5 (1.3)	7.0 (0.8)
Cannabis questionnaires, mean (SD)		
CUDIT-R	11.5 (3.7)	0.1 (0.5)
MCQ-SF	42.1 (15.3)	14.2 (4.4)

LMP = last menstrual period calculated based on participant-reported due date; BMI = body mass index; pregnancy risk factors are for current pregnancy unless otherwise noted. ^a^ data not available for 4 participants (1 miscarriage before measure was administered, 3 dropped out); ^c^ *n* = 31; ^d^ 6/7 of the participants counted as “other” described their race as Hispanic or Latino. A total of 1/6 described their race as “Trinidad-American”, ^e^ *n* = 23, ^f^ *n* = 26. ^h^ Exercise coded as yes if participants reported ≥75 min/week of vigorous activity or ≥150 min/week of light-to-moderate activity; BSI GSI = Brief Symptom Inventory Global Severity Index; PUQE = Pregnancy -Unique Quantification of Emesis; CUDIT = Cannabis Use Disorder Identification Test; MCQ-SF = Marijuana Craving Questionnaire—Short Form.

**Table 2:** Prenatal cannabis use.

Cannabis Use, *n* = 31	Mean (SD)	Median	Range
1st Trimester Frequency, days/week	6.3 (1.3)	7	3.4–7.0
2nd and 3rd Trimester Frequency, days/week	5.5 (1.9)	6.6	0.6–7.0
THC Consumption, mg/day	198.0 (221.2)	108.5	0.6–745.6
CBD Consumption, mg/day	3.5 (4.3)	1.1	0.6–16.3
THC Potency Flower, %	20.0 (10.4)	20.9	0–58.7
THC Potency Concentrate, %	70.4 (20.9)	75.4	2.4–95.1
CBD Potency Flower, %	1.7 (3.9)	1.1	0–15.4
CBD Potency Concentrate, %	4.3 (14.9)	0.1	0–66.8

THC = delta-9-tetrahydrocannabinol, CBD = cannabidiol.

**Table 3:** Birth outcome measures.

	PCE		CONTROL			
Categorical Birth Outcome Measures	*n* ^b^	Mean (SD)	*n* ^b^	Mean (SD)	Cohen’s *d*	*p* Value ^c^
Miscarriage	31	1 (3)	33	0 (0)	N/A	0.48
Stillbirth	31	0 (0)	33	1 (3)	N/A	>0.99
Sex of infant, male	27	13 (48)	32	16 (50)	N/A	>0.99
Sex of infant, female	27	14 (52)	32	16 (50)	N/A	>0.99
Small for Gestational Age, <10th percentile	26	1 (4)	31	0 (0)	N/A	0.46
Low Birth Weight, <2500 g	26	1 (4)	31	0 (0)	N/A	0.46
Preterm birth, <37 weeks	27	0 (0)	31	1 (3)	N/A	0.53
NICU admission ^a^	25	4 (16)	31	1 (3)	N/A	0.16
**Continuous Birth Outcome Measures**						
Gestational age at birth, weeks	28	39.7 (1.2)	31	39.7 (1.2)	0.001	0.97
Weight at birth, grams	26	3277.9 (493.8)	31	3465.1 (326.0)	−0.46	0.09
Birth length, cm	26	49.7 (2.3)	31	50.9 (2.5)	−0.51	0.06
Birth head circumference, cm	25	34.2 (1.6)	30	34.3 (1.3)	−0.08	0.91
Apgar score at 1 min	24	8.2 (0.7)	29	8.1 (0.7)	0.1	0.74
Apgar score at 5 min	24	8.9 (0.3)	29	9.0 (0.3)	−0.42	0.14
Weight at birth, growth percentile	26	38.4 (24.6)	31	51.8 (22.8)	−0.57	0.04
Length at birth, growth percentile	26	39.7 (26.9)	31	55.4 (26.4)	−0.59	0.03
Head circumference at birth, growth percentile	25	42.2 (30.2)	30	42.4 (28.4)	−0.004	0.99

NICU = neonatal intensive care unit; ^a^ the control infant spent 24 h in the NICU, and PCE infants spent 192, 36, 10, and 6 h in the NICU; ^b^ incomplete categorical data received for 4 PCE and 1 CON and incomplete continuous data received for 5 PCE and 3 CON; ^c^ Fisher’s Exact Test for categorical data.

**Table 4:** Birth outcome measures for high-exposure newborns ^a^.

	PCE		Control		Cohen’s *d*	*p* Value
	*n*	Mean (SD)	*n*	Mean (SD)		
Gestational age at birth, weeks	25	39.7 (1.2)	31	39.7 (1.2)	0.01	0.97
Weight at birth, grams	23	3 215.5 (383.0)	31	3 465.1 (236.0)	−0.71	0.01
Birth length, cm	23	49.5 (2.2)	31	50.9 (2.5)	−0.58	0.04
Birth head circumference, cm	22	34.1 (1.4)	30	34.3 (1.3)	−0.14	0.61
Apgar score at 1 min	22	8.2 (0.7)	29	8.1 (0.7)	0.18	0.52
Apgar score at 5 min	22	8.9 (0.4)	29	9.0 (0.3)	−0.45	0.12
Weight at birth, growth percentile	23	35.2 (21.7)	31	51.8 (22.8)	−0.74	0.01
Length at birth, growth percentile	23	37.9 (27.3)	31	55.4 (26.4)	−0.65	0.02
Head circumference at birth, growth percentile	22	40.1 (29.3)	30	42.4 (28.4)	−0.08	0.78

^a^ Mother used cannabis a minimum of three days per week on average throughout pregnancy.

**Table 5:** Sex differences in birth outcomes and cannabis use.

Female Infants	PCE		CONTROL		Cohen’s *d*	*p* Value
	n	Mean (SD)	n	Mean (SD)		
Gestational age at birth	14	39.9 (1.1)	16	39.4 (1.5)	0.44	0.24
Weight at birth, grams	13	3163.2 (354.7)	16	3486.8 (396.2)	−0.86	0.03
Birth length, cm	13	49.5 (2.1)	16	50.2 (3.1)	−0.25	0.50
Birth head circumference, cm	12	33.5 (1.3)	16	34.1 (1.5)	−0.45	0.25
Apgar score at 1 min	12	8.4 (0.7)	15	8.1 (0.6)	0.43	0.28
Apgar score at 5 min	12	8.9 (0.3)	15	9.0 (0.4)	−0.24	0.53
Weight at birth, growth percentile	13	32.6 (19.8)	16	62.9 (22.0)	−1.44	<0.01
Length at birth, growth percentile	13	39.1 (27.1)	16	53.9 (30.9)	−0.51	0.19
Head circumference at birth, growth percentile	12	28.3 (22.7)	16	47.1 (32.4)	−0.65	0.10
**Male Infants**	**PCE**		**CONTROL**		**Cohen’s *d***	***p* Value**
	***n***	**Mean** (**SD**)	***n***	**Mean** (**SD**)		
Gestational age at birth	13	39.4 (1.4)	15	40.0 (0.7)	−0.51	0.19
Weight at birth, grams	13	3392.5 (594.7)	15	3442.1 (241.7)	−0.11	0.77
Birth length, cm	13	49.8 (2.5)	15	51.6 (1.4)	−0.92	0.02
Birth head circumference, cm	13	34.9 (1.5)	14	34.5 (0.9)	0.38	0.33
Apgar score at 1 min	12	7.9 (0.7)	14	8.1 (0.7)	−0.22	0.58
Apgar score at 5 min	12	8.8 (0.4)	14	9.0 (0.0)	−0.63	0.12
Weight at birth, growth percentile	13	44.2 (28.2)	15	39.9 (17.3)	0.19	0.63
Length at birth, growth percentile	13	40.4 (27.7)	15	56.9 (21.6)	−0.67	0.09
Head circumference at birth, growth percentile	13	55.1 (31.3)	14	37.0 (23.0)	0.66	0.10
**Sex Differences, PCE Only**	**MALE**		**FEMALE**		**Cohen’s *d***	***p* Value**
	***n***	**Mean** (**SD**)	***n***	**Mean** (**SD**)		
Gestational age at birth	13	39.4 (1.4)	14	39.9 (1.1)	−0.40	0.30
Apgar score at 1 min	12	7.9 (0.7)	12	8.4 (0.7)	−0.75	0.08
Apgar score at 5 min	12	8.8 (0.4)	12	8.9 (0.3)	−0.24	0.56
Weight at birth, growth percentile	13	44.2 (28.2)	13	32.6 (19.8)	0.48	0.24
Length at birth, growth percentile	13	40.4 (27.7)	13	39.1 (27.1)	0.04	0.90
Head circumference at birth, growth percentile	13	55.1 (31.3)	12	28.3 (22.7)	0.97	0.02
Cannabis use in the first trimester (days/week) ^a^	13	6.0 (1.4)	14	6.7 (0.8)	−0.61	0.13
Cannabis use in the 2nd and 3rd trimester (days/week) ^b^	13	5.0 (1.9)	14	5.9 (1.8)	−0.48	0.22
THC consumption (mg/day) ^c^	13	150.2 (208.3)	14	206.9 (216.8)	−0.27	0.50
CBD consumption (mg/day) ^c^	13	2.1 (2.4)	14	5.5 (5.4)	−0.80	0.05

^a^ The 1st trimester only, measured with the Timeline Follow-Back method. ^b^ Measured by both the Timeline Follow-Back method and weekly diary reports verified with labels. ^c^ Measured by weekly diary reports verified with labels. THC = delta-9-tetrahydrocannabinol; CBD = cannabidiol.

## Data Availability

Restrictions apply to the datasets. Study investigators do not have permission to share data with investigators who were not involved in the original study.

## Supplementary Materials

The following supporting information can be downloaded at https://www.mdpi.com/article/10.3390/children11121436/s1, Table S1: Family Information Form; Table S2 M&M Pregnancy History.

## Appendix A

**Table A1:** Demographic characteristics of PCE participants by sex of newborn.

	Male, *n* = 13	Female, *n* = 14
Socioeconomic status, mean (SD)		
Maternal age at LMP, years	28.9 (4.2)	28.4 (2.8)
Maternal education, years	13.9 (2.4)	13.6 (1.8)
Annual household income, USD	59,838 (37,380)	62,486 (38,578)
Gravidity, n	2.5 (2.8)	2.8 (2.0) ^a^
Pre-pregnancy BMI	31.9 (9.6)	32.6 (10.5) ^a^
Race and Ethnicity, No. (%)		
Hispanic	5 (38)	2 (14)
Caucasian	8 (62)	11 (79)
More than one race	1 (8)	0 (0)
African American	1 (8)	0 (0)
Asian	1 (8)	0 (0)
Other ^b^	2 (15)	2 (14)
Declined to respond	0 (0)	1 (7)
Pregnancy Risk Factors ^a^, No. (%)		
Lack of prenatal care	0 (0)	0 (0)
Hypertension	1 (8)	1 (8)
Preeclampsia	0 (0)	0 (0)
Gestational diabetes	2 (15)	1 (8)
Hyperemesis gravidarum	1 (8)	5 (42)
Previous preterm births	1 (8)	0 (0)
Placental abruption	0 (0)	0 (0)
Exercise ^c,d^, No. (%)		
First trimester, yes	8 (67)	8 (73)
Second trimester, yes	7 (58)	11 (100)
Third trimester, yes	4 (33)	9 (82)
Medication and Supplements, No. (%)		
Prenatal vitamins or folic acid	11 (85)	14 (100)
Prescription antiemetic medication use ^e^	5 (38)	11 (79)
Mental Health and Well Being, mean (SD)		
BSI GSI T-score	55.2 (8.4)	62.7 (5.8)
Perceived Stress Scale Total	19.8 (9.6)	20.1 (5.9)
PUQE	5.2 (1.8)	6.6 (1.5)
Sleep, hours per night	6.4 (1.2)	6.2 (1.1)
Cannabis Questionnaires, mean (SD)		
CUDIT-R	11.0 (2.9)	12.4 (4.5)
MCQ-SF	15.4 (4.3)	12.6 (5.4)

LMP = calculated last menstrual period based on participant-reported due date; pregnancy risk factors are for current pregnancy unless otherwise noted; ^a^ *n* = 12; ^b^ 3/4 of the participants counted as “other” described their race as Hispanic or Latino. In total, 1/4 described their race as “Trinidad-American”; ^c^ *n* = 12 Male; *n* = 11 Female; ^d^ exercise coded as yes if participants reported ≥75 min/week of vigorous activity or ≥150 min/week of light-to-moderate activity; ^e^ *n* = 5 Male; *n* = 11 Female; BSI GSI = Brief Symptom Inventory Global Severity Index; PUQE = Pregnancy Unique Quantification of Emesis; CUDIT = Cannabis Use Disorder Identification Test; MCQ-SF = Marijuana Craving Questionnaire—Short Form.

**Table A2:** Average daily THC and CBD exposure segregated by product format.

Participant	THC mg/d				CBD mg/d			
	Total	Concentrate	Flower	Edible	Total	Concentrate	Flower	Edible
1	745.6	695.0	50.6	0.0	3.2	3.2	0.0	0.0
2	744.2	707.2	35.5	1.4	7.9	7.7	0.1	0.0
3	571.5	0.0	571.5	0.0	7.4	0.0	7.4	0.0
4	525.4	525.4	0.0	0.0	7.2	7.2	0.0	0.0
5	522.2	10.9	511.3	0.0	1.7	0.0	1.7	0.0
6	437.9	0.0	437.9	0.0	1.0	0.0	1.0	0.0
7	397.9	0.0	397.9	0.0	0.4	0.0	0.4	0.0
8	235.8	0.0	234.2	1.6	16.2	0.0	15.2	1.0
9	227.0	132.5	94.4	0.0	0.8	0.6	0.2	0.0
10	219.1	0.0	219.1	0.0	1.1	0.0	1.1	0.0
11	195.3	195.3	0.0	0.0	0.6	0.6	0.0	0.0
12	186.4	11.5	172.6	2.2	3.2	1.7	1.5	0.0
13	170.9	2.0	168.9	0.0	0.1	0.0	0.1	0.0
14	137.8	136.2	1.6	0.0	8.8	8.4	0.3	0.0
15	127.7	0.0	127.3	0.4	0.4	0.0	0.4	0.0
16	108.5	107.1	0.0	1.4	1.5	0.1	0.0	1.4
17	107.0	106.4	0.0	0.6	0.5	0.5	0.0	0.0
18	92.9	43.8	49.0	0.1	0.9	0.7	0.1	0.1
19	88.4	88.4	0.0	0.0	0.1	0.1	0.0	0.0
20	83.7	0.0	77.5	6.3	11.7	0.0	6.0	5.7
21	62.7	2.5	60.2	0.0	3.0	2.4	0.2	0.4
22	45.7	2.1	43.6	0.0	0.9	0.0	0.9	0.0
23	33.5	8.8	24.7	0.0	0.1	0.0	0.1	0.0
24	24.3	0.0	24.3	0.0	0.1	0.0	0.1	0.0
25	18.8	0.7	18.1	0.0	11.6	10.4	1.2	0.0
26	8.6	8.6	0.0	0.0	3.2	3.2	0.0	0.0
27	6.1	2.9	3.2	0.1	0.2	0.0	0.0	0.2
28	5.4	0.0	0.0	5.4	5.8	0.0	0.0	5.8
29	3.3	0.0	3.3	0.0	8.6	0.0	8.6	0.0
30	2.8	0.0	0.1	2.7	0.7	0.0	0.0	0.7
31	0.6	0.0	0.0	0.6	0.6	0.0	0.0	0.6

**Table A3:** Correlations between birth outcome measures and potential confounds.

		Maternal Age at LMP	Years of Education	Household Income	Perceived Stress Scale	Global Severity Index	Gestational Age, Weeks	Birth Weight, g	Birth Length, cm	Birth Head Circumference, cm	Apgar Score at 1 min	Apgar Score at 5 min	Weight at Birth, Growth Percentile	Length at Birth, Growth Percentile	Head Circ. at Birth, Growth Percentile
**CONTROL**															
Maternal Age at LMP	*n*	30	30	30	28	30	30	30	30	29	28	28	30	30	29
	*r*	1	0.33	0.11	0.12	−0.14	−0.22	−0.06	−0.12	0.29	0.26	0.23	0.08	−0.05	0.36
	*p* value		0.07	0.57	0.54	0.45	0.24	0.75	0.55	0.13	0.18	0.23	0.69	0.81	0.06
Years of Education	*n*	30	33	33	31	33	31	31	31	30	29	29	31	31	30
	*r*	0.33	1	0.31	0.01	−0.24	−0.18	0.00	0.05	0.40 *	0.07	0.13	0.16	0.18	0.51 **
	*p* value	0.07		0.09	0.95	0.17	0.34	0.98	0.78	0.03	0.71	0.50	0.39	0.33	<0.01
Household Income	*n*	30	33	33	31	33	31	31	31	30	29	29	31	31	30
	*r*	0.11	0.31	1	−0.21	−0.31	−0.10	−0.12	0.04	0.34	0.02	0.29	−0.08	0.05	0.35
	*p* value	0.57	0.09		0.26	0.08	0.58	0.54	0.85	0.06	0.92	0.12	0.69	0.77	0.06
Perceived Stress Scale	*n*	28	31	31	31	31	29	29	29	28	28	28	29	29	28
	*r*	0.12	0.01	−0.21	1	0.39 *	0.11	−0.05	−0.35	−0.21	0.08	−0.20	−0.09	−0.40 *	−0.26
	*p* value	0.54	0.95	0.26		0.03	0.58	0.80	0.06	0.28	0.70	0.31	0.63	0.03	0.18
Global Severity Index	*n*	30	33	33	31	33	31	31	31	30	29	29	31	31	30
	*r*	−0.14	−0.24	−0.31	0.39 *	1	0.00	0.16	−0.24	−0.14	0.26	−0.08	0.28	−0.25	−0.09
	*p* value	0.45	0.17	0.08	0.03		1.00	0.40	0.19	0.47	0.17	0.70	0.12	0.17	0.65
**PCE**															
Maternal Age at LMP	*n*	27	27	27	25	27	27	25	25	25	24	24	25	25	25
	*r*	1	0.36	0.41 *	−0.35	−0.15	0.13	0.03	−0.18	−0.16	0.15	0.18	−0.06	−0.26	−0.23
	*p* value		0.06	0.04	0.08	0.47	0.51	0.88	0.39	0.44	0.50	0.41	0.79	0.22	0.27
Years of Education	*n*	27	27	27	25	27	27	25	25	25	24	24	25	25	25
	*r*	0.36	1	0.11	−0.15	−0.27	0.04	−0.13	−0.27	0.11	0.15	−0.30	−0.13	−0.32	0.14
	*p* value	0.06		0.58	0.47	0.18	0.83	0.53	0.20	0.59	0.48	0.15	0.55	0.12	0.50
Household Income	*n*	27	27	31	26	31	28	26	26	25	24	24	26	26	25
	*r*	0.41 *	0.11	1	−0.51 **	−0.44 *	0.06	−0.10	−0.17	−0.09	−0.31	−0.38	−0.11	−0.23	−0.10
	*p* value	0.04	0.58		<0.01	0.01	0.77	0.64	0.42	0.66	0.15	0.07	0.60	0.26	0.62
Perceived Stress Scale	*n*	25	25	26	26	26	25	23	23	23	22	22	23	23	23
	*r*	−0.35	−0.15	−0.51 **	1	0.63 **	0.04	0.10	0.15	−0.03	0.18	−0.07	0.11	0.16	−0.05
	*p* value	0.08	0.47	<0.01		<0.01	0.87	0.64	0.48	0.90	0.43	0.74	0.63	0.47	0.81
Global Severity Index	*n*	27	27	31	26	31	28	26	26	25	24	24	26	26	25
	*r*	−0.15	−0.27	−0.44 *	0.63 **	1	−0.08	−0.13	0.07	−0.31	0.26	0.07	−0.02	0.20	−0.23
	*p* value	0.47	0.18	0.01	<0.01		0.69	0.54	0.73	0.13	0.22	0.76	0.93	0.34	0.27

* Pearson’s Correlation significant at the 0.05 level (2-tailed), ** Pearson’s Correlation significant at the 0.01 level (2-tailed), LMP = last menstrual period.
