# Supplementary material for: High-Potency Prenatal Cannabis Exposure and Birth Outcome Measures
Source: Children (Basel). 2024 Nov 26;11(12):1436. doi: 10.3390/children11121436 (PMC11674839; doi:10.3390/children11121436)
Supplement: Supplementary file 1 [file children-11-01436-s001.zip › children-3327188-supplementary.pdf]

Supplemental Material S1

M+M Study
Participant ID: ___________
Date: _____________________
Completed by: _____________
(relationship)

Family Information Form (FIF)

A. GENERAL FAMILY INFORMATION

Our funding agencies require that we note information about education level, employment and household income from the families who participate in our research. We may have asked these questions in the past, and now we would like to follow up to see if there have been any changes since last time. You are free not to answer any question; refusing to answer a question will not affect your ability to participate in this project.

1. **What are your and your child's father's highest levels of education? Please indicate below.**

**Level of School Completed**	**Mother** *(check one)*	**Father** *(check one)*	**Other Primary Guardian** *Specify:________*	**Other Primary Guardian** *Specify: _______*
Less than 7th grade				
Some Junior High/Middle School				
Some High School				
High School Graduate				
Some College				
College Graduate				
Graduate Degree				

2. **What are your and your child's father's current occupations?**

Mother's occupation: ________________________________

Father's occupation: _________________________________

Other primary guardian's occupation: ______________________ (relationship to child) ______________________ (occupation)

Other primary guardian's occupation: ______________________ (relationship to child) ______________________ (occupation)

3. **Please estimate your current annual household income.**

$___________________

B. MOTHER'S MEDICATION USE AND TOBACCO USE

1. **Did you or anyone living in your household smoked tobacco products over the past 12 months? YES NO**
   a. **Who used the tobacco products:** __________________________
   b. **How often:** ____**(> 5 times over 12 months, exclude)**
2. **(For controls) Did you or anyone living in your household use cannabis products by smoking or vaping during or after your pregnancy? YES NO**

Supplemental Material S1

M+M Study
Participant ID: ___________
Date: ______________________
Completed by: _____________
(relationship)

a. **If yes, did the person in your household use the cannabis products inside your household or while in direct contact with you? YES NO**
b. **How often: ____ (> 5 times over 12 months, exclude)**

3. We'd like to get information on the kinds of medications you have taken and/or currently take for behavioral and/or emotional reasons. Do you currently take medications to control depression, anxiety, hyperactivity or anything that is prescribed by a psychiatrist that might be called a "psychiatric" medication?
YES NO

What about in the past? YES NO

If yes, please find each medication in the list below and complete information on reason, dates taken and whether or not he/she is currently taking the medication. If a medication is not in the list, please write in the name and information at bottom of table.

**Name of medicine** *(please circle)*	**Name of med – Generic** *(please circle)*	**Med Class**	**Reason**	**Dates taken** *(Mo/Yr – Mo/Yr)*	**Currently taking?** *(YES/ NO)*
Catapres	Clonidine	Adrenergic			
Tenex	Guanfacine	Adrenergic			
Symmetrel	Amantadine	Anti-Parkinsonian			
Depakote	Valproate	Anticonvulsant			
Dilantin	Dilantin	Anticonvulsant			
Lamictal	Lamotrigine	Anticonvulsant			
Phenobarbital	Phenobarbital	Anticonvulsant			
Tegretol	Carbamazepine	Anticonvulsant			
Topamax	Topiramate	Anticonvulsant			
Trileptal	Oxcarbazepine	Anticonvulsant			
Zonegran	Zonisamide	Anticonvulsant			
Celexa	Citalopram	Antidepressant (SSRI)			
Effexor	Venlafaxine	Antidepressant (SSRI)			
Lexapro	Escitalopram	Antidepressant (SSRI)			
Luvox	Fluvoxamine	Antidepressant (SSRI)			
Paxil	Paroxetine	Antidepressant (SSRI)			
Prozac	Fluoxetine	Antidepressant (SSRI)			
Zoloft	Sertraline	Antidepressant (SSRI)			
Imipramine	Imipramine	Antidepressant (Tricyclic)			
Desyrel	Trazodone	Antidepressant (other)			
Abilify	Aripiprazole	Antipsychotic			
Geodon	Ziprasidone	Antipsychotic			
Risperdal	Risperidone	Antipsychotic			
Seroquel		Antipsychotic			
Zyprexa	Olanzapine	Antipsychotic			
Mellaril	Thioridazine	Antipsychotic			
Valium	Diazepam	Benzodiazepine Anxiolytics			
Xanax	Alprazolam	Benzodiazepine Anxiolytics			
Buspar	Buspirone	Other Anxiolytic			
Melatonin	Melatonin	Melatonin			
Strattera	Atomoxetine	Norepinephrine Re-Uptake Inhibitor			

Supplemental Material S1

M+M Study
Participant ID: ___________
Date: ______________________
Completed by: _____________
(relationship)

**Name of medicine** *(please circle)*	**Name of med – Generic** *(please circle)*	**Med Class**	**Reason**	**Dates taken** *(Mo/Yr – Mo/Yr)*	**Currently taking?** *(YES/ NO)*
Cyproheptadine	Cyproheptadin	Other Psychotropic			
Adderall	Amphetamine	Stimulant			
Focalin	Dexmethylphenidate	Stimulant			
Ritalin	Methylphenidate	Stimulant			

4. Do you currently take medications other than for behavioral and/or emotional reasons (and the medication[s] to help control seizures you had mentioned earlier)? YES NO
(***If YES,*** *specify below. Also list any seizure medications from above.)*

What about in the past? YES NO
*(**If YES,** specify below)*

**Name of medicine**	**Name of med – Generic**	**Med Class**	**Reason**	**Dates taken** *(Mo/Yr – Mo/Yr)*	**Currently taking?** *(YES/ NO)*

Supplemental Material S2

ID# ________________ Date: ________________

Interviewer Name: ______________________________

M&M Pregnancy History

1. Height: ______ feet ______ inches
2. Pre-pregnancy weight: _______ lbs
3. How many previous pregnancies have you had? ________
4. How far apart were your pregnancies, from birth to conception? _______ months
5. Where are you receiving prenatal care?

   ☐ Obstetrician ☐ Family physician ☐ Midwife ☐ No prenatal care ☐ Other:

Instructions: For each item, please mark if you have experienced it during this pregnancy, a previous pregnancy, or if you know of a family member who has experienced it. For family members, please specify the relationship to you.

6. Hypertension (high blood pressure) in pregnancy ☐ This pregnancy ☐ Previous pregnancy ☐ Family member:
7. Preeclampsia ☐ This pregnancy ☐ Previous pregnancy ☐ Family member:
8. Gestational diabetes ☐ This pregnancy ☐ Previous pregnancy ☐ Family member:
9. Hyperemesis gravidarum ☐ This pregnancy ☐ Previous pregnancy ☐ Family member:
10. Preterm birth ☐ This pregnancy ☐ Previous pregnancy ☐ Family member:
11. Placental abruption ☐ This pregnancy ☐ Previous pregnancy ☐ Family member:
